# Simple and affordable soft brace application in dystrophic epidermolysis bullosa patients

**DOI:** 10.3389/fsurg.2023.1189962

**Published:** 2024-01-03

**Authors:** Chong Wu, Xin-He Jiao

**Affiliations:** ^1^ The Fifth Clinical Medical College of Henan University of Chinese Medicine (Zhengzhou People’s Hospital), Zhengzhou, Henan, China; ^2^ Department of Plastic Surgery, The Second Affiliated Hospital of Zhengzhou University, Zhengzhou, Henan, China

**Keywords:** epidermolysis bullosa, surgical prognosis, hand function, influencing factors, soft brace

## Abstract

**Background:**

Dystrophic epidermolysis bullosa (DEB) is a hereditary disease characterized by increased fragility of the epidermis and mucosa and is accompanied by blister formation following minor trauma. Repeated injuries cause contracture and scar formation, which can further result in hand deformity, leading to a decline in hand ability and a lower quality of life. In this study, after the scar release of patients' hands, we developed a new and practical portable soft support, and evaluated its efficacy in delaying the scar contracture of hands after operation.

**Methods:**

According to the hand function scores, the patients were divided into two groups. Those with excellent and good grades were assigned to the open hand function group, and those with poor grades were allocated to the restricted hand function group. The primary conditions, the use of a postoperative soft brace, and some common factors in the two groups were compared to determine whether these parameters influence postoperative hand function.

**Results:**

There were no significant differences in age, gender, body mass index, ADL assessment index, albumin concentration, hemoglobin concentration, fasting blood glucose level, prothrombin time, and activated partial thromboplastin time between the two groups (*p* > 0.05). In contrast, there was a significant difference between the two groups in the use of soft braces following the operation (*p* < 0.05). The odds ratio of patients fixed with a brace compared with patients not fixed with soft braces was 11.01.

**Conclusions:**

Soft brace is a critical factor impacting the hand function of patients with dystrophic epidermolysis bullosa after scar contracture release in both hands. Indeed, a hand brace worn after the operation can delay the recurrence of scar contracture in both hands and offer patients a longer time to use their hands effectively. In addition, by restoring the appearance of patients' hands and some hand functions, patients' mental state and quality of life have been greatly improved.

## Introduction

Dystrophic epidermolysis bullosa (DEB) is a genetic disease characterized by increased skin fragility and blisters after minor trauma. It is distinguished by blisters under the thick plate (1). In patients with DEB, continuous use of hands may indeed increase the risk of scar contractures and deformities. The literature notes that DEB patients often present with flexion contractures in their hands, primarily due to scarring and contraction following repetitive trauma. These scars can lead to limited functionality in fingers and hands, resulting in deformities such as mitten deformities and pseudosyndactyly (2). In epidermolysis bullosa patients, scar contracture deformity of both hands mainly occurs in DEB (3). If the patient suffers from bilateral scar contracture deformity, the only treatment option is to perform bilateral scar contracture release (4). However, new blisters are formed on the hands owing to the daily use of the hands. Postoperative recurrence is common in both hands. Previous studies have established that recurrence occurs every two years (5). Nevertheless, we hope patients get the maximum treatment benefit after the operation. Therefore, we followed up with patients after the operation and evaluated the progress of postoperative hand function limitations according to the evaluation standard (6) formulated by the hand surgery branch of the Chinese Medical Association in 2000. The possible influencing factors after operation were statistically studied, and the effects of these factors on hand function after operation were analyzed, aiming to identify factors affecting the recurrence of hand scar contracture in DEB patients to achieve a better prognosis and quality of life.

In the past, some scholars have recommended that patients use braces after the operation, but no specific evidence supports the fact that braces can limit the recurrence of scar contracture. In this study, we made a recommended soft brace for the patients. This type of soft braces consists of erythromycin eye ointment, growth factor (not used after discharge), vaseline gauze, and gauze bandages. The braces not only prevents friction between fingers but also offers good breathability and is simple to make. This soft brace not only isolated the skin friction in daily life, but also had a certain anti-infection effect. In view of the cumbersome and time-consuming nursing methods, only some patients' hands were well cared for after the operation. Considering that postoperative hand function limitations of patients may be related to postoperative care, it was also included in the analysis. Studying the influencing factors of postoperative hand function can provide interventions to prevent the recurrence of scar contracture in both hands of DEB patients and maximize the therapeutic effect of surgery. Therefore, in this study, we produced a novel soft brace and analyzed the potential role of the soft brace in delaying the development of hand contracture in patients with DEB.

## Materials and methods

### Data collection

A total of 43 patients who had been effectively followed up were included. Among them were 20 males (46.5%) and 23 females (53.5%). Females outnumbered males by 1.15 times, with ages ranging from 2 to 28 years old. Given the patients developed the disease shortly after birth, the duration of the disease was the same as their age. The operation times were 1–3. Baseline demographics such as age, gender, nationality, body mass index (BMI), the ability of daily living assessment index (Barthel Index), albumin concentration (ALB), hemoglobin concentration (Hgb), fasting blood glucose (Glu) level, prothrombin time (PT), activated partial thromboplastin time (APTT) were collected from the medical record system.

### Inclusion and exclusion criteria

**Figure 1:**
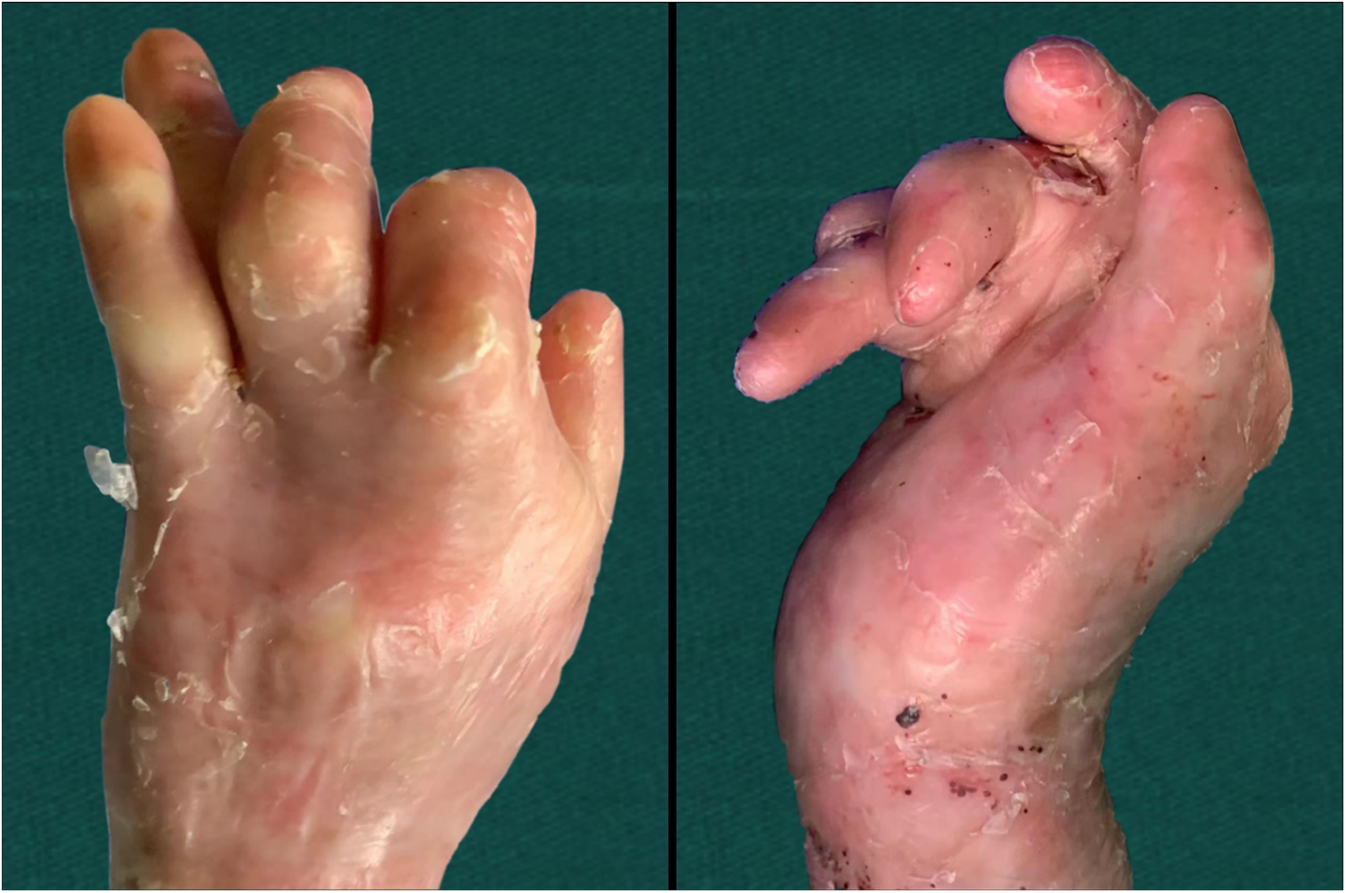
Preoperative photographs.

The inclusion criteria were as follows: patients with visible scar contracture deformity in both hands (Figure 1); patients requiring surgical treatment and signed the informed consent form for surgery; patients without a history of severe complications and who were eligible for surgery. The exclusion criteria were as follows: patients with major underlying diseases, complicated with severe malnutrition or anemia that have not been corrected; patients not indicated for surgery; patients who were followed up for less than two years.

In addition, we included patients who did not use a soft brace at home every day in the not used braces group, and patients who used a brace at home into the use soft braces group.

### Surgical technique

Following admission, the patients actively improved after various preoperative examinations and could be operated on after no contraindications were identified. The patients and their families were offered thorough preoperative education to inform them of the possibility of postoperative parenteral nutritional support and blood transfusion. Caution was taken to avoid daily rough contact with the skin, which could result in blister formation. A liquid or semi-liquid diet was recommended to prevent solid food from damaging the digestive tract of patients. Reasonable feeding methods and good feeding quality can optimize patients' growth and enhance gastrointestinal tract function, immune status, and wound healing abilities (7).

**Figure 2:**
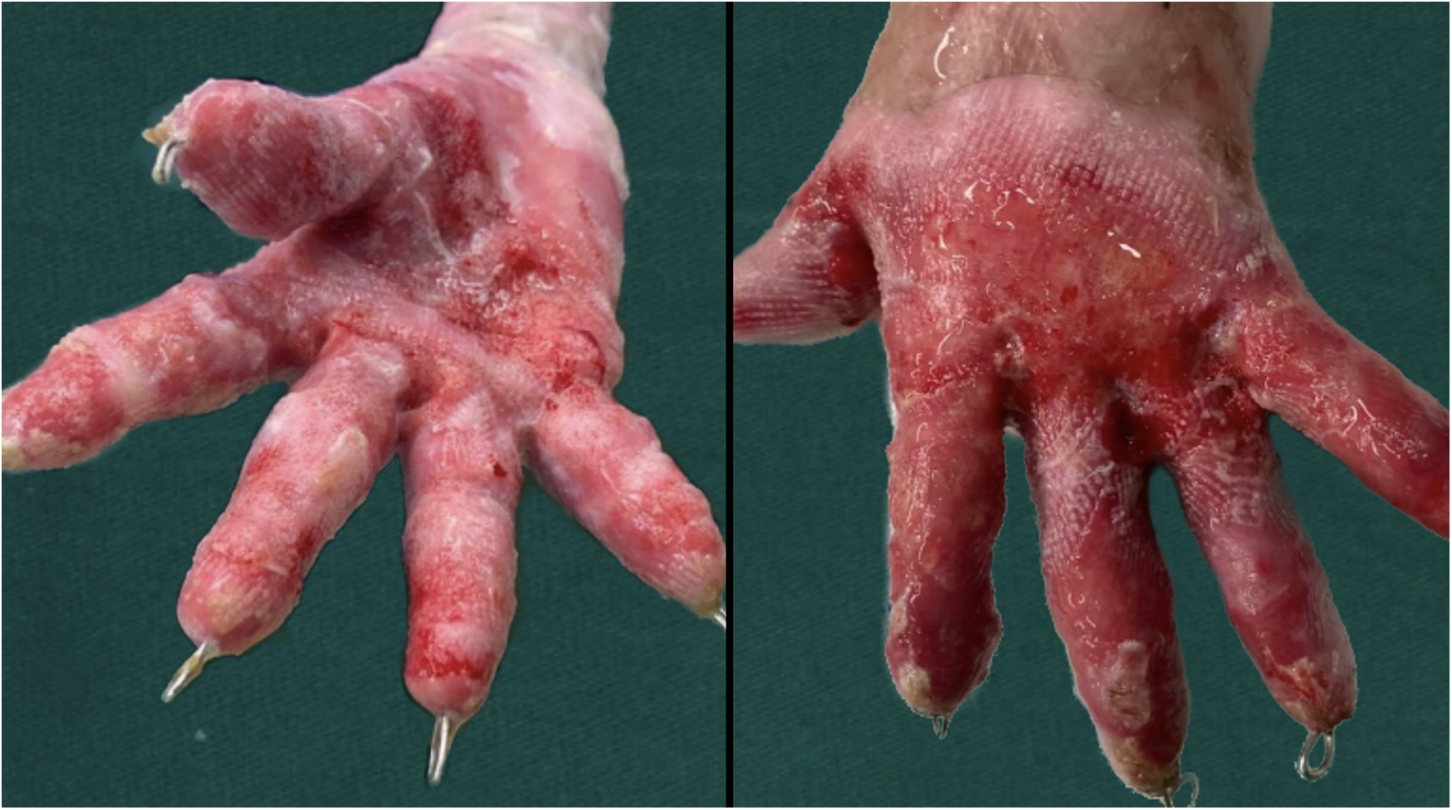
Palmar and dorsal photos of the patient after the first dressing change.

Considering the fragile respiratory mucosa of DEB patients, endotracheal intubation was not performed. All DEB patients underwent deep induction anesthesia and local infiltration anesthesia using a mask for oxygen inhalation and without endotracheal intubation. Adhesions between each finger and the palm of the patient with palm scar contracture were separated, and the outer skin was sliced longitudinally along the skin adhesion. The contracture was relieved so that the fingers could be passively straightened. Each finger was then fixed with Kirschner wires, which can only pass through the interphalangeal joint but not the metacarpophalangeal joint (8). The fingers were subsequently kept straight, rinsed with iodophor solution, hydrogen peroxide, and normal saline, wrapped with hydrophilic oil yarn and gauze, and the inner gauze was soaked with epidermal growth factor solution. A hydrophilic oil gauze was utilized to prevent direct contact between the gauze and wound, reduce the adhesion between gauze and skin, and reduce pain caused by dressings after the operation. Finally, the loose gauze strips were layered, and sufficient pressure was applied. Afterward, the hands were wrapped with an elastic bandage. The ends of the fingers were monitored to observe the blood supply in the fingers. After the operation, the dressing was changed on a weekly basis (Figure 2). During dressing change, all dressings were removed, washed repeatedly with a large amount of iodophor solution, hydrogen peroxide, and normal saline, and the hands were wrapped with an elastic bandage. With the growth of the epidermis of both hands, each dressing change can gradually reduce the use of dressings. Given that the long-term fixation of Kirschner wires may lead to infection and joint injury, it was removed after three dressing changes.

### Post-operative management

**Figure 3:**
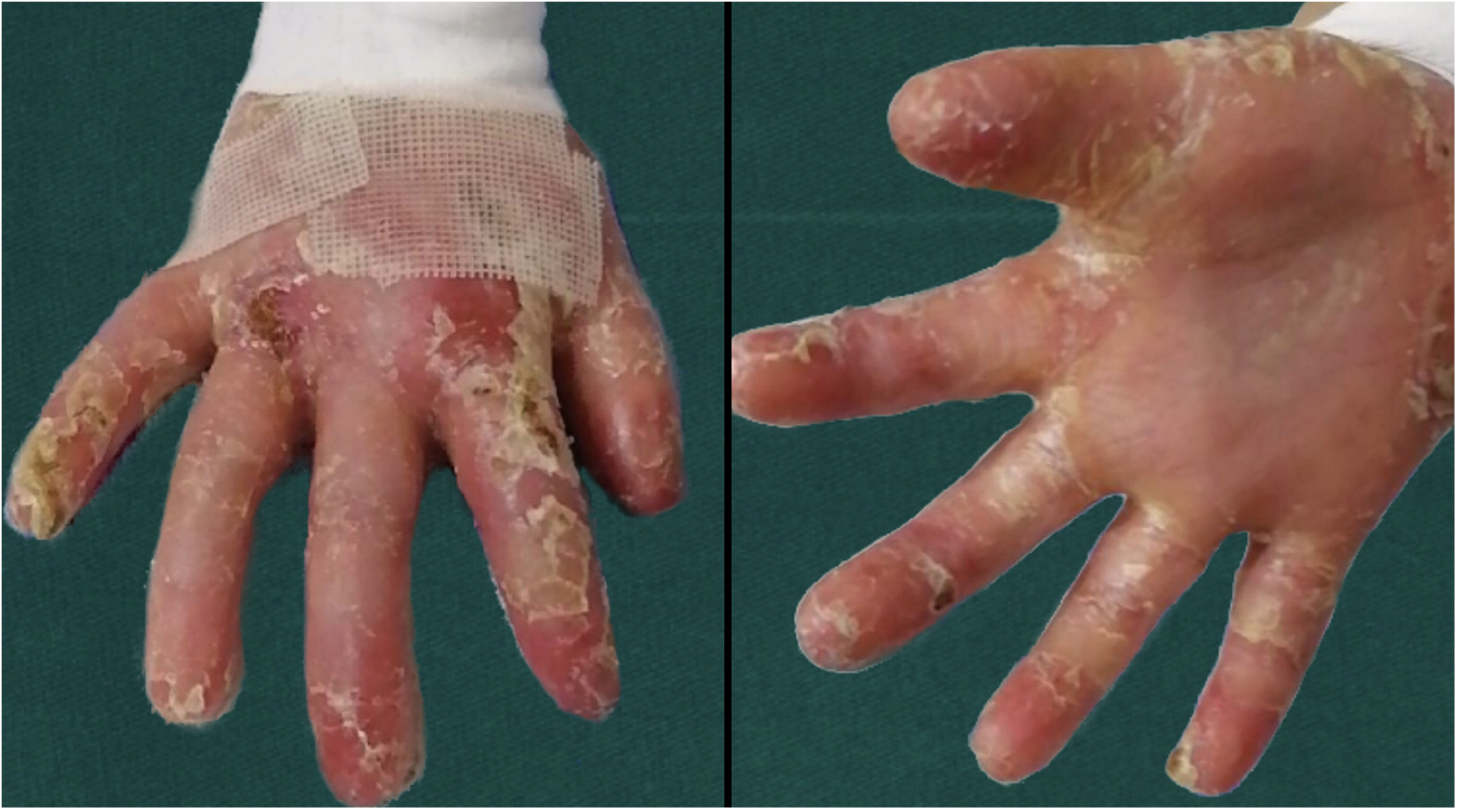
Dorsal and palmar photos of the patient 3 month after operation.

After discharge, patients were encouraged to exercise their hand function as soon as possible. DEB patients used soft braces daily at night and during daytime rest. They were urged to practice fine movements, such as writing and dressing. As is well documented, exercise can enhance hand function (9). At the same time, braces were used to separate each finger, with the fingers slightly abducted, to prevent the formation of new blisters and scar adhesion. Light clothing was recommended to avoid injury caused by friction between the rough and rugged clothes and the skin (Figure 3). When applying dressings, care was taken to avoid accidental shedding of dressings, resulting in adhesion between the damaged skin, clothes, and other items. When changing dressings, attention was paid to avoid skin damage caused by direct tearing of the dressings. In addition, the postoperative psychological counseling for patients is also extremely important.

In this study, we assessed hand function every 3 months in patients with DEB after surgery. Data were collected via telephone follow-up and a questionnaire survey, and the use of hand braces after the operation was tracked. In this study, the motor function of both hands, activities of daily living, sensory recovery, blood circulation, appearance, and recovery of work were recorded three years after the operation. According to the evaluation standard (6) of the hand surgery branch of the Chinese Medical Association, patient hand function was divided into four grades: excellent, good, poor, and inferior (Supplementary Table S1). Patients whose hand function was evaluated as excellent and good were allocated to the open hand function group, whereas patients whose hand function was evaluated as poor and poor were assigned to the restricted hand function group.

### Statistical analysis

SPSS 26.0 was used to analyze the factors affecting postoperative hand function. The Shapiro-Wilk (S-W) test was used to determine if the data followed the normal distribution. Data such as body mass index and ADL assessment index were expressed as mean ± standard deviation and compared by the *t*-test, whereas data such as gender were expressed in percentage and compared using test. Logistic regression analysis was employed to compare the influencing factors between the two groups. *P* < 0.05 was considered statistically significant.

## Results

### General data of two groups of patients

A total of 43 patients without missing follow-up information were divided into groups according to the hand function evaluation criteria. There were 22 patients in the restricted hand function limited group and 21 in the open hand function group.In our results, there was a significant difference in the use of two-hand braces between the two groups (*p* = 0.0004) (Table 1). Although the *p*-value was not statistically significant (*p* = 0.091), there was some difference in the number of male and female in each group, so we included gender in the Logistics regression analysis for further study to explore its effect on postoperative hand function.

**Table 1:** General data (counting data) of two groups of patients.

Factor	The restricted hand function group (*n *= 22) (%)	The open hand function group (*n *= 21) (%)	*x* ^2^	*p*
Gender			2.87	0.091
Male	59.09 (*n *= 13)	33.33 (*n *= 7)		
Female	40.90 (*n *= 9)	66.67 (*n *= 14)		
Usage of brace			12.44	0.0004
Used braces	72.73 (*n *= 16)	19.05 (*n *= 4)		
Not used braces	27.27 (*n *= 6)	80.95 (*n *= 17)		

### Comparison of clinical indicators in different subgroups

There was no significant difference in age, gender, body mass index, life ability assessment index, albumin concentration, hemoglobin concentration, fasting blood glucose level, prothrombin time, and activated partial thromboplastin time between the two groups (*p* > 0.05) (Table 2). In contrast, soft brace is a critical factor impacting the hand function of patients with dystrophic epidermolysis bullosa after scar contracture release in both hands (*p* < 0.05) (Table 1). In addition, we included bimanual brace use in the Logistics regression analysis for further analysis.

**Table 2:** General data (counting data) of two groups of patients.

Factor	The restricted hand function group (*n *= 22) (*Mean *± *SD*)	The open hand function group (*n *= 21) (*Mean *± *SD*)	*t*	*p*
Age (year)	7.50 ± 5.25	8.62 ± 6.18	−0.64	0.525
The operation times	1.14 ± 0.35	1.24 ± 0.54	−0.74	0.466
BMI(kg/m^2^)	19.65 ± 5.21	19.79 ± 5.71	−0.09	0.929
BARTHEL index	62.73 ± 9.60	61.43 ± 7.10	0.50	0.618
ALB(g/l)	38.13 ± 5.61	35.36 ± 8.27	1.29	0.205
HGB(g/l)	95.41 ± 19.62	99.19 ± 20.36	−0.62	0.538
GLU(mmol/l)	4.44 ± 0.70	4.26 ± 0.53	0.95	0.346
PT(s)	12.23 ± 1.14	12.77 ± 2.36	−0.96	0.345
APTT(s)	38.56 ± 4.47	40.73 ± 6.73	−1.25	0.218

**Figure 4:**
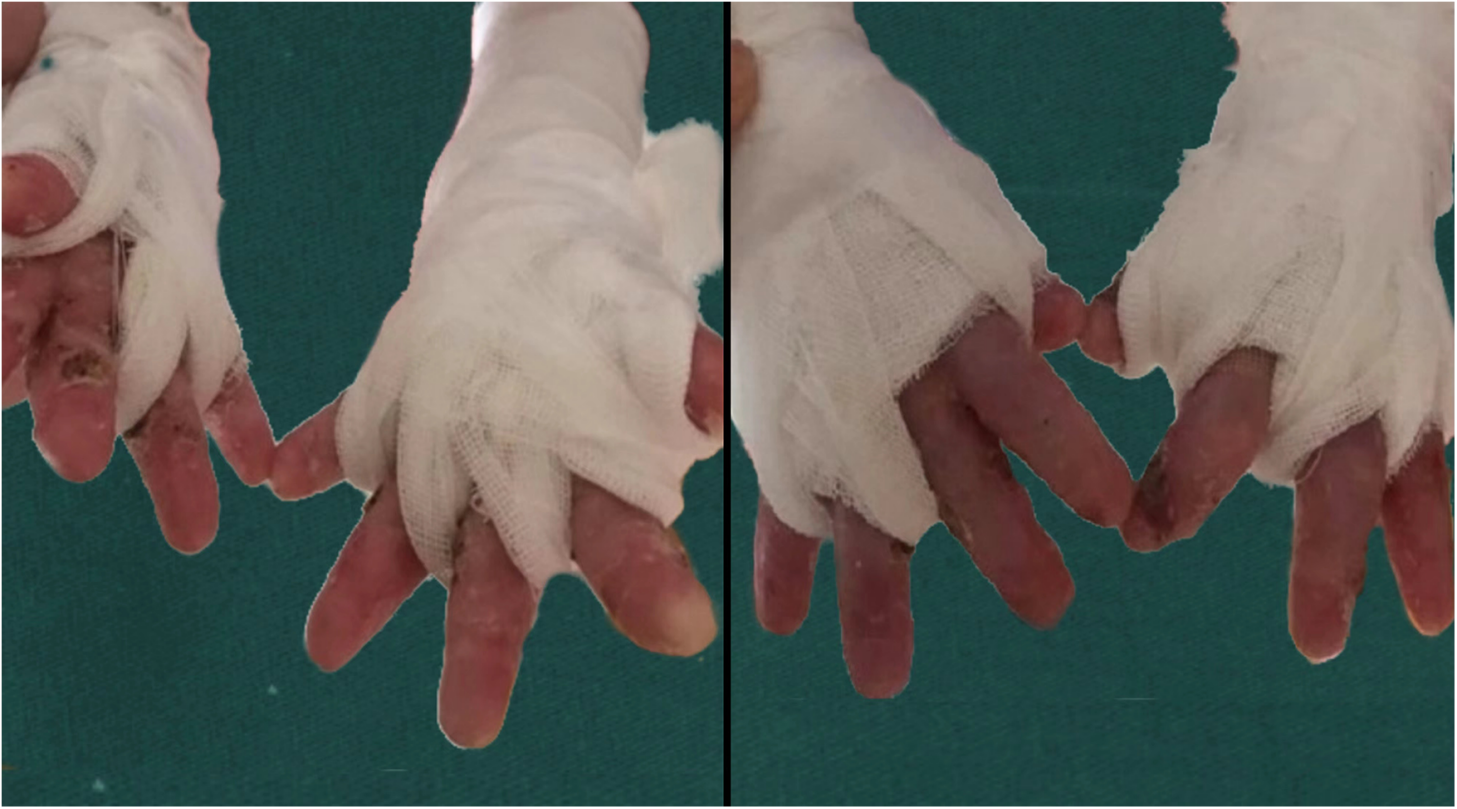
Soft brace fixation.

Finally, our results exposed that a postoperative soft brace is an independent influencing factor of whether the patient's hand function was limited after the operation (Table 3). Under identical treatment conditions, the time to postoperative hand function limitation was longer in patients with brace fixation, and the odds ratio of patients with brace fixation compared with patients without brace fixation was 11.01. Compared with the rigid brace proposed by Jennifer et al. (9), the soft braces we adopted can better prevent the damage caused during the finger movement (Figure 3). Figure 4 illustrated the portable soft brace used in this study.

**Table 3:** Logistic analysis of factors affecting postoperative hand function of patients.

Factor	*OR*	95%*CI*	*P*
Gender	2.71	0.63,11.71	0.181
Usage of brace	11.01	2.52,48.19	0.001

Finally, the average interval between reoperations was 30.39 months in the use soft braces group and 19.5 months in the not used braces group. This suggested that the use of soft braces can delay the onset of scarring contracture in the hand.

## Discussion

Due to the low prevalence of DEB, there are relatively few studies on the recurrence of scar contracture and deformity in both hands caused by DEB. However, whether the patients achieve the expected improvement in quality of life after the surgery is a great concern. Therefore, this study analyzed factors that may influence the postoperative hand function of patients, hoping to limit scar contracture by controlling factors that affect postoperative hand function to prolong the adequate usage time of the hands brought about by the operation. More importantly, comparing the data between the open hand function group and the restricted hand function group revealed that the use of braces in the two groups had a significant statistical difference in postoperative hand function. Using a brace to fix both hands after the operation can effectively delay secondary scar contracture in both hands and extend the time to hand function limitation. Furthermore, age, gender, body mass index, life ability assessment index, albumin concentration, hemoglobin concentration, fasting blood glucose level, prothrombin time, and activated partial thromboplastin time had no significant effect on postoperative hand function. Given that there was a potential difference in gender between the two groups, it was included in the logistic regression analysis as a factor that may affect the postoperative hand function of patients. Although the final result was not statistically significant (*p* > 0.05), Deng et al. observed that female patients had more severe phenotypes in gene mutations and speculated that there might be a modified gene that played a phenotypic effect in autosomal-inherited DEB (10). Thus, the effect of gender on the postoperative hand function of DEB patients warrants further investigations.

DEB patients usually develop blisters and ulcerated wounds all over the body, impacting their quality of life (11). Due to the frequent use of hands in daily life, DEB patients often have severe hand deformities owing to repeated blistering and ulceration, resulting in a deterioration in the quality of life and the inability to meet the most basic daily needs. At present, fibroblasts, mesenchymal stem cells, and protein replacement have shown promising efficacy in improving DEB symptoms. However, they cannot cure DEB (12). Reconstructing the patients' hands through surgical manipulation is crucial for patients reintegrating into society (13). Herein, the patients were treated with bilateral scar contracture release, and the fingers were fixed with Kirschner wires without skin grafting, in line with the treatment modality employed by Diedrichson et al. (4). The surgery predominantly restored fine movements such as grasping and pinching and substantially enhanced the patient's ability to take care of themselves. This operation is safe and effective and can achieve favorable surgical results. However, with the formation of new blisters on the patient's hands after the operation, adhesion may occur after wound healing, resulting in scar contracture, which will eventually develop into a “glove hand” deformity. During the follow-up of the patients, we found that scar contracture deformity recurred in varying degrees in both hands, with recurrence being more noticeable in non-profit hands (14). Scar contracture deformity of both hands will not only lead to muscular atrophy but also may influence the development of patients' bones, resulting in considerable limitations in everyday self-care abilities.

Our study uncovered that after the operation, the patients' hands were covered with hydrophilic oil dressings on the finger seams, palms, and other friction-prone parts, wrapped with gauze and bandages, with each finger separated and ensuring a certain degree of abduction, which could effectively prevent blister formation and adhesion after healing. We recommend that patients use soft braces to separate the fingers during the day or use dynamic orthosis for fixation and thermoplastic orthosis to separate and fix fingers at night. During the day, soft braces should be used to wrap and separate the fingers, and continuous hand function exercises should be performed to promote the input of somatic sensation and the recovery of hand function (15). Patients should use the brace to fix their hands for at least 8 h at night if they are not using it during the day. In a study conducted by Xianyu et al., the hands of patients were fixed with thermoplastic splints from 3 weeks to 3 months after the operation, which achieved promising results (8).

However, follow-up data demonstrated that only a subset of patients had their hands fixed with braces. The main reason is that as the disease progresses, some clinical symptoms of DEB patients, including blisters on the skin of the whole body, mucosal ulcers, etc., gradually worsen with time (16), making everyday care more challenging. DEB patients who eventually develop systemic diseases die of squamous cell carcinoma (17, 18). The patient's family members are unwilling to invest too much time and money, resulting in insufficient postoperative hand care. Therefore, The invention of a simple and practical soft support has become an important problem. In this study we developed a brand new practical portable soft brace. The wound was covered with an oily dressing and a gauze was subsequently bandaged to prevent further adhesion of the tissue. As its incidence is multi-system, further treatment also requires the cooperation of a multidisciplinary team (19), as well as the support of the family and society. In addition, psychological counseling after hand function reconstruction is also extremely important. DEB patients often fall into anxiety due to their unusual appearance. Therefore, appropriate psychological counseling can alleviate the patient's anxiety, which may be beneficial to the patient's prognosis. However, further research is needed to test this hypothesis. The soft brace used in this study has the following advantages: (1) cheap; (2) convenient replacement; (3) The extension of hand contracture after surgery is conducive to patients' mental health and helps patients to face life and disease with a positive attitude.

This study mainly analyzed the situation of DEB patients, the use of postoperative braces, and other possible influencing factors. Factors related to drug therapy, adherence, and educational attainment still necessitate further studies. As the sample size was small herein, the number of cases included in the analysis was low, which may have impacted the results. We hypothesize that using a hand brace after surgical treatment of DEB hand scar contracture deformity is an essential factor affecting the postoperative hand function of patients. Using a hand brace will help prolong the recurrence interval of scar contracture in both hands and enable patients to obtain a better prognosis and quality of life.

## Conclusion

Through the analysis of the baseline demographics and postoperative follow-up data of the included patients, our study determined that age, gender, nationality, body mass index, life ability assessment index, albumin concentration, hemoglobin concentration, fasting blood glucose level, prothrombin time and activated partial thromboplastin time of patients with DEB had no significant effect on postoperative hand function. Besides, the application of soft braces after the release of scar contracture of both hands in patients with DEB is an essential factor affecting postoperative hand function. Finally, the use of a postoperative hand brace can delay the recurrence of postoperative scar contracture in both hands and provide the patients more time to use their hands effectively.

## Data Availability

The original contributions presented in the study are included in the article/**Supplementary Material**, further inquiries can be directed to the corresponding author.
